# Tomato Plants Treated with Systemin Peptide Show Enhanced Levels of Direct and Indirect Defense Associated with Increased Expression of Defense-Related Genes

**DOI:** 10.3390/plants8100395

**Published:** 2019-10-03

**Authors:** Mariangela Coppola, Ilaria Di Lelio, Alessandra Romanelli, Liberata Gualtieri, Donata Molisso, Michelina Ruocco, Concetta Avitabile, Roberto Natale, Pasquale Cascone, Emilio Guerrieri, Francesco Pennacchio, Rosa Rao

**Affiliations:** 1 Dipartimento di Agraria, Università degli Studi di Napoli Federico II, Via Università 100, 80055 Portici, Italy; mariangela.coppola@unina.it (M.C.); ilaria.dilelio@unina.it (I.D.L.); donata.molisso@unina.it (D.M.);; 2 Dipartimento di Scienze Farmaceutiche, Università degli Studi di Milano, via Venezian 21, 20133 Milano, Italy; alessandra.romanelli@unimi.it; 3 CNR-IPSP, Via Università 133, 80055 Portici, Italy; liberata.gualtieri@ipsp.cnr.it (L.G.); michelina.ruocco@ipsp.cnr.it (M.R.);; 4 CNR-IBB, Via Mezzocannone 16, 80134 Napoli, Italy; connieavitabile@libero.it

**Keywords:** biopesticide, endogenous defenses, insect herbivores, phytopathogenic fungi, parasitoids, tomato protection

## Abstract

Plant defense peptides represent an important class of compounds active against pathogens and insects. These molecules controlling immune barriers can potentially be used as novel tools for plant protection, which mimic natural defense mechanisms against invaders. The constitutive expression in tomato plants of the precursor of the defense peptide systemin was previously demonstrated to increase tolerance against moth larvae and aphids and to hamper the colonization by phytopathogenic fungi, through the expression of a wealth of defense-related genes. In this work we studied the impact of the exogenous supply of systemin to tomato plants on pests to evaluate the use of the peptide as a tool for crop protection in non-transgenic approaches. By combining gene expression studies and bioassays with different pests we demonstrate that the exogenous supply of systemin to tomato plants enhances both direct and indirect defense barriers. Experimental plants, exposed to this peptide by foliar spotting or root uptake through hydroponic culture, impaired larval growth and development of the noctuid moth *Spodoptera littoralis*, even across generations, reduced the leaf colonization by the fungal pathogen *Botrytis cinerea* and were more attractive towards natural herbivore antagonists. The induction of these defense responses was found to be associated with molecular and biochemical changes under control of the systemin signalling cascade. Our results indicate that the direct delivery of systemin, likely characterized by a null effect on non-target organisms, represents an interesting tool for the sustainable protection of tomato plants.

## 1. Introduction

The use of synthetic pesticides has significantly fostered the success of modern agriculture, but has concurrently shown that their abuse generates a number of ecological, environmental and health problems. Growing awareness of these critical issues in public opinion and among policy makers was the background that a decade ago led to the definition of EU directive (2009/128) on sustainable use of pesticides. The consequent need to reduce pesticide use in agriculture has considerably promoted research efforts aiming to discover new plant protection tools with low impact on the environment and non-target organisms. These research efforts have increasingly shed light on the mechanisms underlying antagonistic interactions in nature, offering the opportunity to use the molecular weapons adopted by the fighting organisms, shaped by a long co-evolutionary history. We can define this approach as learning from nature to develop bio-inspired strategies of pest management [1,2].

The detection of invading organisms is a crucial step in plant immunity, which initiates the activation of defense responses. Herbivore-associated elicitors (HAE) are molecules recognized by the plant, each selectively inducing different segments of the defense reaction pathways [3]. Anti-herbivore defenses are induced not only by molecules produced by the invading organisms, but also by endogenous plant molecules, that are released upon damage caused by pest insects and pathogens and, therefore, are commonly referred as damage-associated molecular patterns (DAMP) [4]. These molecules, act as warning signals [5,6,7]. The effective amplification of this signal and of the triggered defense responses is under control of enzymatic cascades, which are up-regulated by the feeding damage. For example, reactive oxygen species (ROS) signals are produced by nicotinamide adenine dinucleotide phosphate (NADPH) oxidase [8], while cell wall fragments originate in the activity of polygalacturonase; both enzymes are induced by mechanical wounding or by biotic stress agents [9,10], which concurrently induce downstream genes encoding the precursors of endogenous peptide elicitors [7,11]. Peptides are the smallest biological molecules of the plant proteome that fulfill diverse roles in plant growth, development, reproduction, symbiotic interactions, and stress responses [12,13,14]. For example, Pep1 from *Arabidopsis thaliana* (*Arabidopsis*) was shown to activate defense genes associated with the innate immune response [11]. Systemin (Sys) was the first peptide signal discovered in plants [7]. It is an 18 amino acid peptide, which derives from the carboxy terminus of a 200 amino acid precursor called prosystemin (ProSys). The *prosystemin* gene evolved in species of the Solaneae subtribe of the *Solanaceae* family, including tomato, potato, bell pepper, nightshade, but it is not found in tobacco or *Arabidopsis* [7]. Upon wounding or insect attack, the expression of *ProSys* is increased and the encoded protein precursor is processed to release the Sys peptide, which interacts with a membrane-bound receptor to initiate a complex signalling cascade that leads to the production of defense compounds [15,16,17]. Perception of Sys at the cell surface stimulates cell membrane depolarization, which induces an efflux of K^+^ and influx of Ca^2+^ into the cell [15]. The concurrent activation of a Mitogen-activated protein kinase and calmodulin and of a downstream phospholipase promotes the release of linolenic acid from thylakoid membranes. Linolenic acid is then converted to jasmonic acid (JA) and other oxylipin signals, through the octadecanoid pathway, to regulate the expression of defense-related genes and the synthesis of defense metabolites [15]. Sys and JA appear to contribute to the propagation of the long-distance signal; systemin acts at the site of wounding to trigger the production of JA that, in turn, promotes a long distance defense response [18,19]. The regulation of Sys production and release is still largely unknown, but the enzymatic processing of its precursor appears to be mediated by phytaspases [20]. SR160/BRI1 has previously been postulated as the systemin receptor in tomato [16,21]), a rejected hypothesis recently replaced by the systemin receptor 1 and 2 (SYR1 and SYR 2) proposed by [17].

This defensive cascade has been described for tomato where Sys is not the unique signaling peptide. For example, the hydroxyproline-rich systemin glycopeptides (HypSys) are 18–20 amino acids in length, released from larger precursors, isolated from tomato and other plant species and active in plant defense [22,23]. Sys and HypSys work cooperatively to upregulate the systemic wound defense response in tomato [24].

Moreover, other genetically distinct families of plant defense signal peptides have been described in several species [11,14,22,25,26,27,28]. 

The modulation of direct and indirect defenses exerted by Sys in tomato plants under insect attack has been widely characterized [15,29,30,31,32]. Sys signaling flows into the promotion of direct and indirect defense responses against Lepidoptera larvae, aphids and phytopathogenic fungi [14,30,33] that include the production of protease inhibitors (PIs) and other compounds interfering with herbivore larval growth and survival and fungal colonization of the plant [29,30,34,35]. In addition, the Sys-mediated indirect defenses involve the modification of the composition of the volatile blend emitted by tomato plants with the consequent increase of attractiveness towards herbivore natural antagonists [36,37].

It was previously demonstrated that the constitutive expression of the prosystemin cDNA promotes the up-regulation of an array of defense genes, controlled by different signaling pathways conferring protection against both biotic and abiotic environmental challenges [30,35,38] although the plants showed some differences in phenotypes and physiology as, for example, reduced internodes elongation, delayed flowering time, reduced leaf area and stomatal conductance [38,39]. Thus, a single peptide hormone is capable of eliciting multiple defense pathways to counteract a wide range of unfavourable conditions for the plant. Therefore, the over-expression of *ProSys* in tomato plants is a valuable tool to reduce the loss inflicted by different biotic stressors. However, the continuous activation of the prosystemin gene that are normally induced by pests is costly, affecting the growth and the physiology of tomato plants. To develop an alternative delivery strategy, not relying upon transgenic plants, we investigated the effect of the exogenous application of the Sys peptide on the defense responses and its potential use as a plant protection strategy in tomato. 

Here we demonstrate that Sys-treated plants, by spotting the peptide on intact leaves of healthy plants or by supplying it through hydroponic cultures, are resistant to the noctuid moth *Spodoptera littoralis* (*S. littoralis*) and to the fungal pathogen *Botrytis cinerea* (*B. cinerea*) and show an increased production of volatile compounds able to attract insect natural enemies. The resistant phenotype of treated plants is associated with the expression of an array of defense-related genes induced upon systemin treatment. These results prove that the use of the exogenous supply of Sys to tomato plant represents an interesting approach for the protection of the crop.

## 2. Results

### 2.1. Sys Supply Promotes Direct Defenses against Spodoptera littoralis

**Figure 1:**
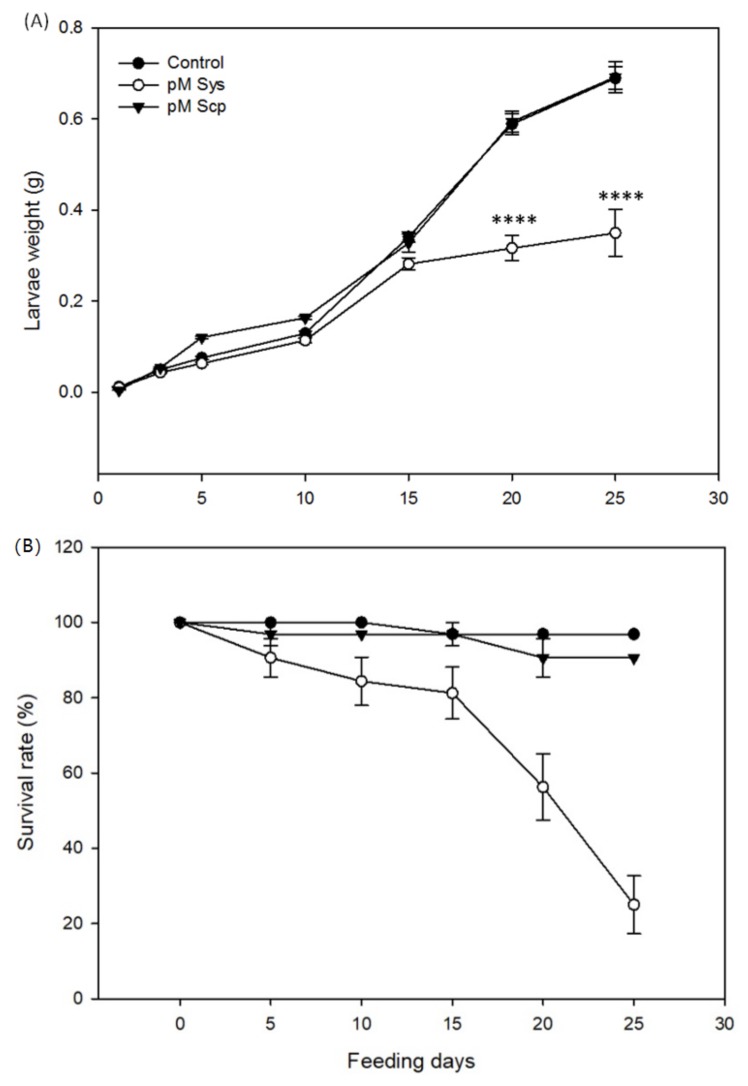
Systemin (Sys) foliar applications on *S. littoralis* larvae. (**A**) Mean weight (± S.E., standard error) of *S. littoralis* larvae feeding on control and treated leaves. (**B**) Survival rate of experimental *S. littoralis* larvae. Asterisks denote statistically significant differences (one-way Analysis of Variance, ANOVA: **** *p* < 0.00001). A group of 32 larvae was used for each experimental condition and the experiment was repeated twice.

In order to assess the impact of Sys supply on the growth and mortality of *S. littoralis* larvae, a feeding bioassay was carried out, by comparing Sys-treated plants with untreated or Scp treated controls. Based on the gene expression results (see below), we decided to use 100 pM Sys solution, and larvae were fed with tomato leaves treated with this concentration of the peptide. The reduced weight gain was already evident after 5 days of feeding, and this consistent trend over time generated significant differences after day 15 (One Way ANOVA test: *p* < 0.0001, *F* = 14.9) (Figure 1A). Moreover, the survival rate of experimental larvae was significantly lower when fed on treated leaves than on controls (Log-rank (Mantel-Cox) test: *p* < 0.0001, *dF* = 2, *χ2* = 51.16) (Figure 1B). After 25 days of feeding, the survival rate was as low as 25% in larvae fed on Sys-treated plants, compared to 90% and 97% for Scp and control plants, respectively (Figure 1B). Thus, Sys foliar application impairs both growth and survival of *S. littoralis* larvae.

**Figure 2:**
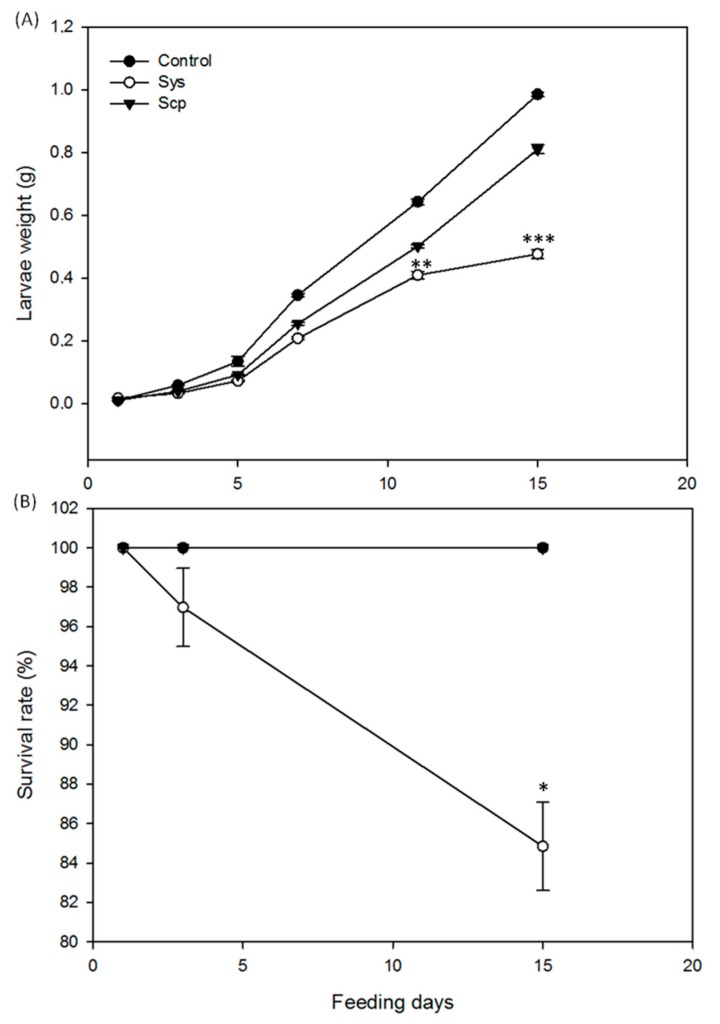
Effect on insect performance of systemin peptide supplied via hydroponics. Tomato plants were grown in hydroponic culture and supplied with 100 pM Sys or 100 pM Sys-scramble (Scp) or PBS1X. (**A**) Mean weight (± S.E.) of *S. littoralis* larvae feeding on tomato leaves. (**B**) Survival rate of experimental *S. littoralis* larvae. Asterisks denote statistically significant differences (one-way ANOVA: * *p* < 0.05; ** *p* < 0.01; *** *p* < 0.001). A group of 32 larvae was used for each experimental condition and the experiment was repeated twice.

Similarly, Sys supply in hydroponic cultures determined negative effects on larval growth and survival (Figure 2). Larvae fed with leaves from tomato plants kept on Sys-enriched hydroponics showed a significant reduction in weight starting 5 days after the onset of the bioassay (one-way ANOVA: *p* < 0.0001, *F*(2.93) = 67.837) (Figure 2A); the survival rate of larvae fed on hydroponics was significantly reduced if compared with the other two control groups (Log-rank (Mantel-Cox) test: *p* < 0.023; *df* = 1; *χ2* = 5.164) (Figure 2B).

**Figure 3:**
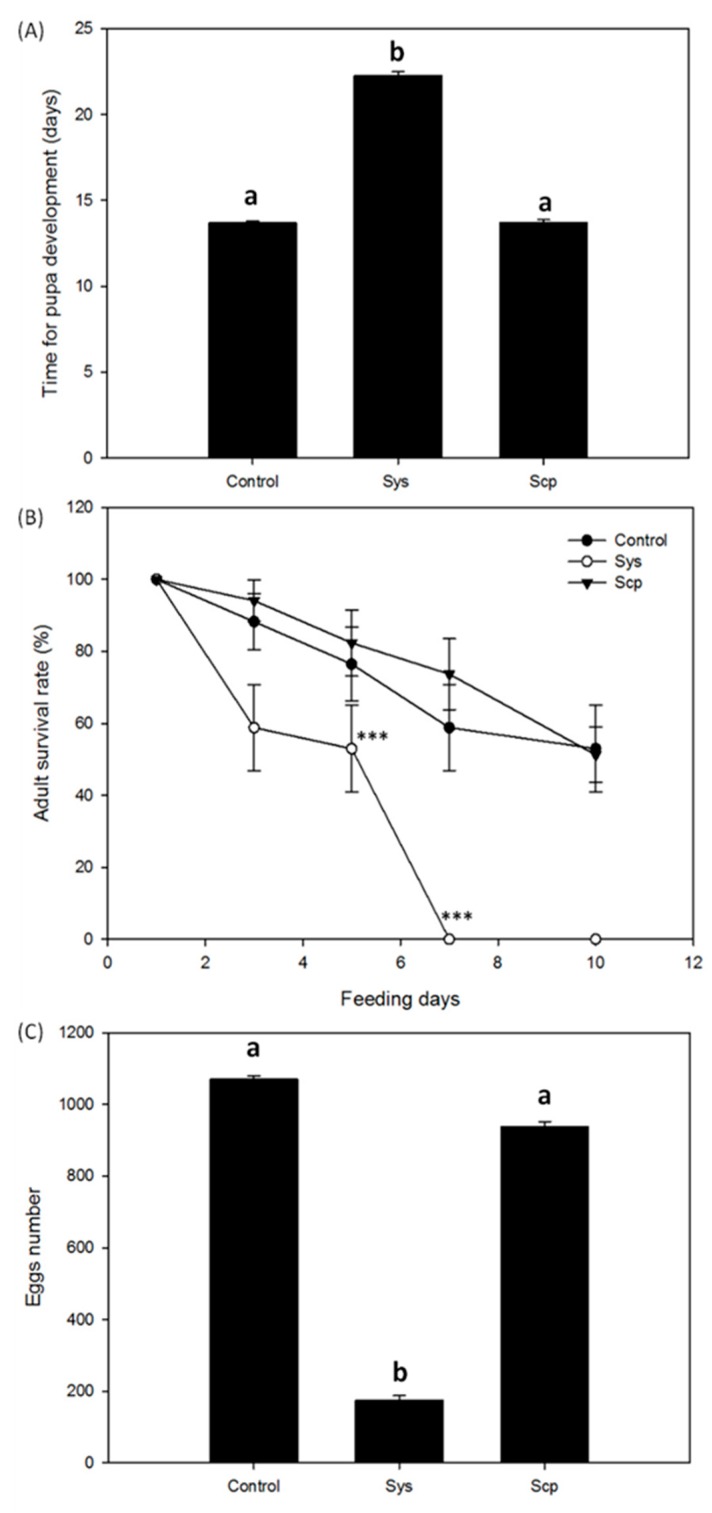
Systemin effect on development and reproduction of *Spodoptera littoralis* larvae. Tomato plants supplied with 100 pM Sys, or 100 pM Scp or PBS1X in hydroponics were used to feed *S. littoralis* larvae, on which the following parameters were scored: duration of pupal development (**A**), adult survival rate (**B**) and number of laid eggs (**C**). Letters and asterisks denote statistically significant differences (*** *p* < 0.001; one-way ANOVA). A group of 32 larvae was used for each experimental condition and the experiment was repeated twice.

The surviving experimental larvae were monitored for pupal development, adult survival and reproduction. Indeed, the time required by the experimental larvae to pupate was significantly higher in Sys treated plants (Kruskal-Wallis Test: *p* < 0.0001; *KW* = 71.170; *n* = 32) (Figure 3A). In addition, the emerged adults showed a significantly reduced survival rate (log-rank (Mantel-Cox) test: *p* < 0.0001, *dF* = 2, *χ2* = 45.04) (Figure 3B) and a significantly lower fecundity (one-way ANOVA test *p* < 0.0001; *F*(2.37) = 37.496) (Figure 3C).

### 2.2. Sys Supply Enhances Plant Tolerance against Botrytis Cinerea

**Figure 4:**
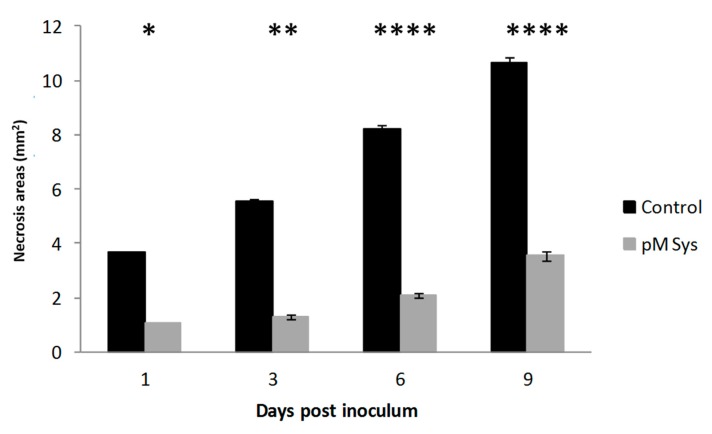
Enhanced resistance to *Botrytis cinerea* of Sys treated leaves. Response to *B. cinerea* infection by leaves of plant treated with 100 pM Systemin. The graphs display the average (± S.D.) of the lesion size at 1, 3, 6 and 9 days post inoculum. Asterisks denote statistically significant differences (T-test: * *p* < 0.05; ** *p* < 0.01; *** *p* < 0.001; **** *p* < 0.00001).

**Figure 5:**
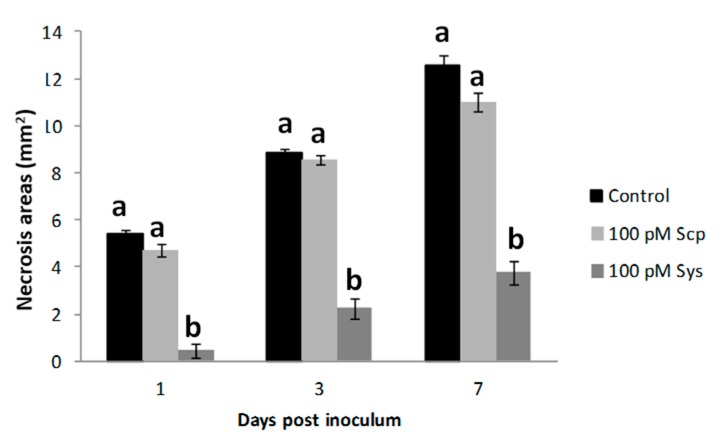
Enhanced resistance to *Botrytis cinerea* of tomato plants supplied with systemin via hydroponics. Response to *B. cinerea* infection by leaves from plants treated with 100 pM Sys, or 100 pM Scp or PBS1X in hydroponics. The graphs display the average (± S.D.) of the lesion size at 1, 3 and 7 days post inoculum. Letters denote statistically significant differences (One-way ANOVA, Tukey test).

Since tomato transgenic lines with over-expression or reduced expression of ProSys showed respectively increased resistance or increased susceptibility to *B. cinerea*, we evaluated the performance of Sys-treated plants against this necrotrophic fungus, at four different time points (1, 3, 6 and 9 days pi). Disease severity was quantified by measuring necrotic areas. Sys-treated leaves displayed a marked reduction of *B. cinerea* induced lesions at all the time points considered (highest significant differences at six and nine days post inoculum with *p* < 0.00001) (Figure 4 and Figure S1), similarly to what observed following the fungal inoculum on plant grown in hydroponic media enriched with the same concentration of Sys (*p* < 0.05) (Figure 5). Hydroponic supply of Scp did not produce any difference with controls. These results demonstrate that the hydroponic supply of the Sys peptide interferes with fungal growth following leaf colonization and reduce disease severity.

### 2.3. Sys Supply Promotes Indirect Defenses by Increasing the Emission of VOCs

**Figure 6:**
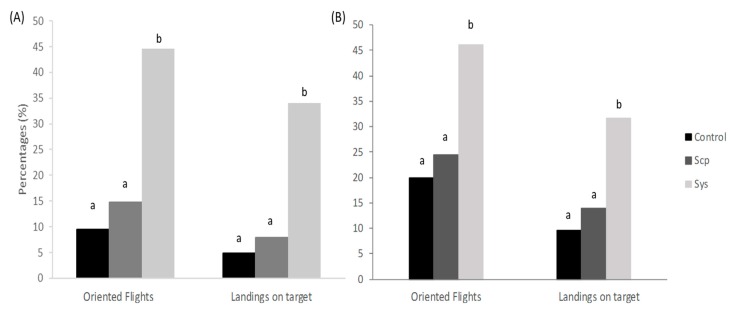
Flight behaviour of the aphid parasitoid *Aphidius ervi* towards tomato plants treated with Sys, Scp, and untreated (control) on intact leaves (**A**) or in hydroponics (**B**). Values indicate the percentage of females showing oriented flights and landings on source. Each assay was conducted using at least 100 females tested against 9 plants. Different letters indicate significant differences (G-test, *p* < 0.05).

Tomato plants treated with Sys on intact leaves showed an increased attractiveness towards *A. ervi* females compared to the control (Figure 6A). *A. ervi* females showed 45% of oriented flights and 40% of landings on Sys-treated plants in comparison to 9.5% (G test, *χ2* = 31.35, *df* = 1, *p* < 0.01) and 4.8% (G test, *χ2* = 27.60, *df* = 1, *p* < 0.01) observed for controls, respectively. Similarly, plants grown in the presence of Sys-enriched hydroponic solution elicited 46.2% of oriented flights and 31.6% of landings on targets in comparison to 20% (G test, *χ2* = 17.01, *df* = 1, *p* < 0.01) and 9.6% (G test, *χ2* = 15.72, *df* = 1, *p* < 0.01) recorded for the controls (Figure 6B). No significant difference in parasitoid attraction was noted for Scp-treated plants, both on leaves and in hydroponics in respect to controls (Figure 6A,B). In order to experimentally support the observed increased attractiveness towards the parasitoid, we analyzed the volatile blend emitted by leaf-treated plants with the experimental peptides with the aim to identify volatile signals known to be involved in indirect defense. Under the described experimental conditions, we registered a quantitative variation in volatile blends released by treated plants (Table 1).

A group of compounds associated with attractiveness towards insect natural enemies (Benzaldehyde, Ethylbenzene, p-Xylene, β-Ocimene, α-pinene, Limonene, Methyl-Jasmonate and β-caryophyllene) were found to be strongly increased (around 10 folds) by Sys application, while no differences were observed for mock- and Scp-treated plants (Table 1). In order to directly address the effect of Sys exogenous supply on the promotion of JA-mediated direct and indirect defenses, the absolute quantification of MeJA was carried out (Figure S2). Sys-treated plants released 2.57 × 10^8^ ppbv of MeJA, significantly higher in comparison to Control and Scp (around 1 × 10^8^ ppbv).

### 2.4. Systemin Supply on Leaves of Intact Plants Induce the Expression of Defense Genes

**Figure 7:**
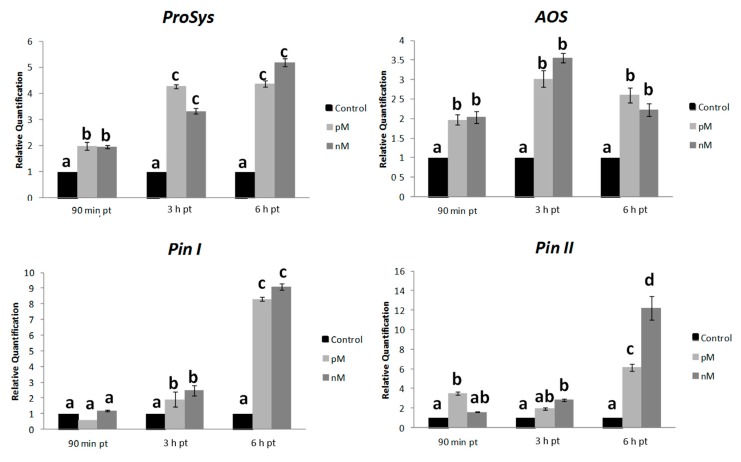
Gene expression analysis in leaf treated with Sys (local). Quantification of transcripts of early (*ProSys, AOS*) and late genes (*Pin I, Pin II*) by Reverse-Transcription-Polymerase Chain Reaction (RT-PCR) after 90 min, 3 h and 6 h following 100 pM and 100 nM systemin peptide treatment. Relative quantities are calibrated on samples obtained from tomato leaves spotted with PBS1X (Control). For each gene, relative quantification (*RQ*) variations have been analysed by two-way ANOVA. Different letters denote significantly different values (*p* < 0.01). Error bars indicate standard error.

**Figure 8:**
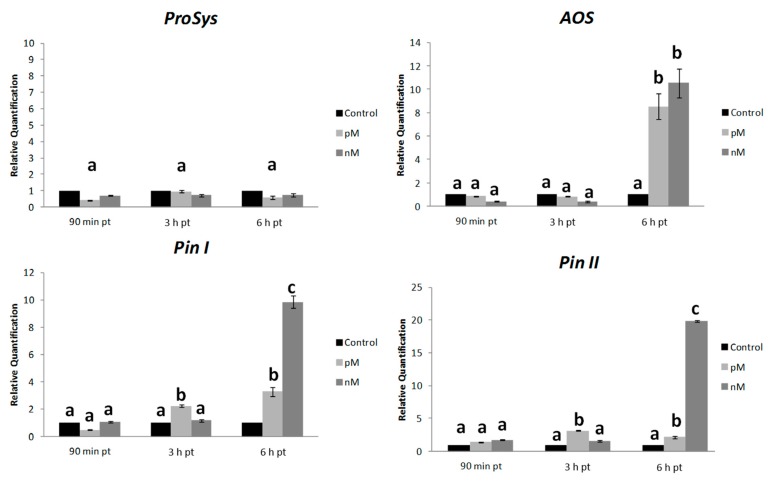
Systemic gene expression analysis in leaves upon Sys foliar treatment. Quantification of transcripts of early (*ProSys, AOS*) and late genes (*Pin I, Pin II*) in leaves distal from the treated ones by real time RT-PCR after 90 min, 3 h and 6 h following 100 pM and 100 nM systemin peptide treatment. Relative quantities are calibrated on samples obtained from tomato leaves spotted with PBS1X (Control). For each gene, *RQ* variations have been analysed by two-way ANOVA. Different letters denote significantly different values (*ProSys*: *p* > 0.05; *AOS*: *p* < 0.01 6 h pt; *Pin I* and *Pin II*: *p* < 0.05 3 h pt, *p* < 0.01 6 h pt). Error bars indicate standard error.

**Figure 9:**
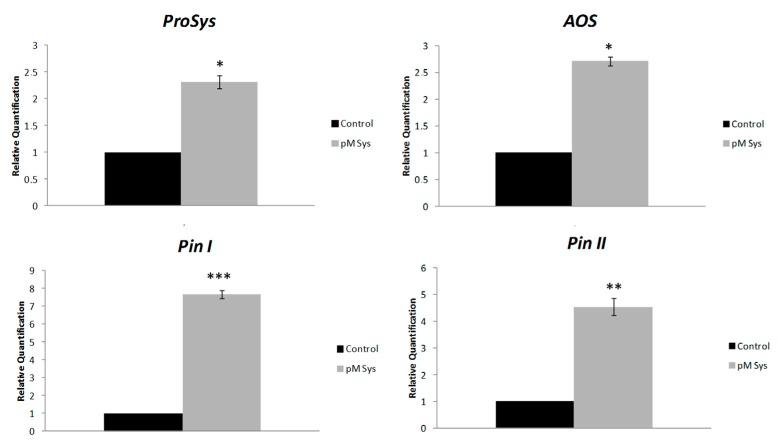
Gene expression in plants grown in hydroponic solution containing Sys. Quantification of transcripts of *ProSys, AOS, Pin I* and *Pin II* by Real Time RT-PCR detected in leaves of plants grown in a hydroponic system, 3 h after the addition of 100 pM systemin. Relative quantities are calibrated on samples obtained from tomato leaves of plant grown in a hydroponic system supplied with PBS1X. Asterisks denote statistically significant differences (* *p* < 0.05, ** *p* < 0.001, *** *p* < 0.0001; T-test). Error bars indicate standard error.

To investigate the effect of the exogenous supply of Sys at molecular level, we monitored the expression of defense-related genes in plantstreated by spotting a Sys solution on the abaxial face of fully expanded healthy leaves or adding the peptide in the hydroponic medium. Then we quantified the transcripts of early (signaling related: *Prosystemin*, *ProSys*, and *Allene Oxide Synthase*, *AOS*) and late (defense-related: *wound-induced proteinase inhibitors I* and *II*, *Pin I* and *Pin II*) genes on Sys- and Scp- treated plants. The expression of target genes was analyzed in a time-course assay by qRT-PCR, on plants exposed to two different concentrations of the experimental peptides. Relative quantification of treated samples was referred to the mock-treated control (relative quantification; *RQ* = 1). An enhanced transcription of the selected genes, both in the treated leaves (Figure 7) and in distal leaves (untreated leaves of treated plants) (Figure 8) was observed. In the treated leaves (Figure 7), *ProSys* transcripts significantly increased and maximal accumulation occurred within 3 h (*F* = 0.0124; *p* = 0.00276), while *AOS* transcripts doubled after 90 min and remained constantly transcribed at higher levels at all experimental time-points. A different transcript profile was observed for *Pin I* (*F* = 0.00813; *p* = 0.00312) and *Pin II* (*F* = 0.047; *p* = 0.00272), which showed a gradual increase, to reach a peak after 6 h. A dose-dependent effect of Sys treatment was observed for *Pin II* transcription after 6 h. In the distal leaves (Figure 8), no *ProSys* transcript up-regulation was observed, while *AOS* transcript greatly increased after 6 h. *Pin I* and *Pin II* transcripts showed a moderate up-regulation after 3 h and a high increase after 6 h. Similarly, to what observed for the expression of the early genes, following the application of the two different Sys concentrations, a different level of expression of the late genes was registered: the 100 pM concentration had the strongest induction effect on the gene transcription. No significant variation in the transcript levels of the tested genes was registered in leaves treated with Scp (Figure S3). Thus, the observed transcriptional enhancement of selected genes is unequivocally associated with the leaf application of the Sys peptide. The same transcripts were monitored in the leaves of plants grown under hydroponics enriched with 100 pM Sys. All the transcripts were significantly up-regulated (*p* value: *ProSys*, *p* = 0.0219; *AOS*, *p* = 0.02037; *Pin I*, *p* = 0.0001; *Pin II*, *p* = 0.0038) (Figure 9), while no significant transcript increase was observed following Scp application (Figure S4). These results demonstrate that hydroponic supply of Sys is able to induce the transcription of defense-related genes associated with the Sys signaling pathway.

## 3. Materials and Methods

Two different peptides were produced: Sys and Sys-scramble (Scp), the latter was used as control. Peptides synthesis, purification and stability are described elsewhere [40]. Briefly, the peptides were obtained by solid phase synthesis following standard protocols [41]. Purification of the peptides was carried out by Reversed-Phase High-Performance Liquid Chromatography (RP-HPLC) (Shimadzu LC-8A, equipped with a SPD-M10 AV) on a semipreparative column (Jupiter 10µProteo 90A, 250 × 10.0 mm, Phenomenex, Torrance, CA, USA) using a gradient of acetonitrile (0.1% TFA) in water (0.1% TFA) from 5 to 50% in 30 min at 5 mL/min. Peptides were characterized by mass spectrometry (LC-MS ESI-TOF 6230 Agilent Technologies, Milan, Italy). Systemin sequence: AVQSKPPSKRDPPKMQTD. Mass calculated: 2009.3 Mass found: 670.94 [M + 3H]^3+^; 1005.60 [M + 2H]^2+^.

Systemin scramble sequence: KSKMDRQPVQAPDKPSPT. Mass calculated: 2009.3 Mass found: 670.96 [M + 3H]^3+^; 1005.53 [M + 2H]^2+^.

Peptide stability was tested as previously described [40]. Analysis of the HPLC (Shimadzu LC-8A, equipped with a SPD-M10 AV) profiles and of the mass spectra collected indicates that the peptide is stable in all the tested conditions [40]. Stock solutions of the synthesized peptides were prepared as described in [42].

### 3.1. Plant Materials

The tomato (*Solanum lycopersicum* L.) cultivar used was “Red Setter”. Seeds were germinated on sterile paper disks moistened with water and kept in the dark for three days in a climate chamber at 24 ± 1 °C. At the break of cotyledons, seeds were exposed to a 16:8 h light:dark photoperiod, for 48 h. Germinated seeds were transferred to sterile soil in a climate chamber, at 26 ± 1 °C, under a 16:8 h light:dark photoperiod. Four weeks-old plants were used for biological and molecular investigations, unless otherwise indicated. Intact leaves were treated with 2 μL of 100 pM and 100 nM Sys or Scp, by spotting the abaxial surface using a pipette. Both peptides were dissolved in phosphate buffer solution (PBS). Control plants were similarly treated with the buffer. Treated leaves (local leaves) were used for the expression analysis and bioassays with pests.

For hydroponics, tomato seeds, at two-cotyledon stage (5 days after sowing), were transferred into a hydroponic system, and grown for 4 weeks in a 5 L solution, containing Mg(NO_3_)_2_·6H_2_O (384.0 mg/L), Ca(NO_3_)_2_·4H_2_O (812.9 mg/L), KNO_3_ (101.5 mg/L), K_2_SO_4_ (319.3 mg/L), KH_2_PO_4_ (204.8 mg/L), Hydromix (14.0 mg/L), and the experimental peptides to a final concentration of 100 pM.

### 3.2. Bioassay with Spodoptera Littoralis

Feeding bioassays with the phytophagous insect *S. littoralis* larvae were carried out as previously described [30]. Briefly, larvae were obtained from a laboratory population maintained at Isagro Ricerca (Novara, Italy) and reared in our laboratory for more than 10 generations, in a climate chamber at 25 ± 2 °C; 70 ± 5% relative humidity (RH); 16:8 h light:dark photoperiod. Larvae were fed with an artificial diet composed as follow: 41.4 g L^−1^ wheat germ, 59.2 g L^−1^ brewer’s yeast and 165 g L^−1^ corn meal, supplemented with 5.9 g L^−1^ ascorbic acid, 1.8 g L^−1^ methyl 4-hydroxybenzoate and 29.6 g L^−1^ agar. Larvae grown on this artificial diet until the 2^nd^ instar. Uniform second instar larvae were selected in groups of 32 individuals, and each group was used to evaluate larval weight and survival rate as affected by the treatment with 100 pM Sys compared to controls mock-treated (phosphate buffer; PBS) or supplied with 100 pM Scp. Every day, leaves from five control or treated plants (biological replicates) were harvested. Similar leaves, in terms of size and position on the plant, were used to produce leaf disks to feed experimental larvae. Tomato leaf disks were laid down on 2% agar (*w/v*) to create a moist environment required to keep them turgid in a tray well (Bio-Ba-32, Color-Dec, Lucca, Italy) covered by perforated plastic lids (Bio-Cv-4, Color-Dec, Lucca, Italy). Larvae were singly separated into each box and fed with the correspondent leaf disk (control or treated). These were daily replaced, adjusting the size (initially of 2 cm^2^, later of 3, 4 and 5 cm^2^) in order to meet the food needs of growing larvae. Plastic trays were incubated at controlled conditions (28 ± 1 °C; 70 ± 5% RH; 16:8 h light:dark photoperiod). Larval weight and mortality were recorded until pupation, which took place into plastic boxes containing vermiculite (25 × 10 × 15 cm).

For Sys supplied in hydroponics, the 3^rd^ instar larvae were used for which larval weight and longevity were registered. In addition, the following reproduction-related parameters were recorded: time for pupa development (from the onset of the bioassay to pupation), adult longevity and fecundity. Briefly, pupae were collected, washed in a 50% solution of bleach (0.05% sodium hypochlorite), rinsed with distilled water and air dried, before they were sexed under a stereomicroscope (40×) by observing morphological characters, as described [43], separated in aerated plastic boxes (25 × 10 × 15 cm) and daily inspected until adult emergence. After emergence, adults had access to a 50% water solution of honey. Males and females were kept together (1 female:2 males) for 24 h, at 25 °C, to allow mating. Then, mated females were separated from males (marked with red ink) and singly transferred into a plastic cylinder (diameter 8 cm, height 9 cm), lined with paper where their egg laying activity was assessed on a daily basis, for the whole lifespan, by counting the number of eggs deposited on paper, under a stereomicroscope operating at 40× magnification. Longevity of the adults was also recorded. Each experiment was repeated two times.

### 3.3. Bioassay with Botrytis Cinerea

Four week-old plants, treated with 100 pM Sys directly delivered on the leaf surface or dissolved in the hydroponic solution (final Sys concentration was 100 pM), were tested for resistance to *B. cinerea*. Spores of the fungus were obtained as follow: suspension in sterile distilled water, filtration through sterile Kimwipes (Kimberly-Clark) to remove fragments of hyphae, and adjustment to a concentration of 1·10^6^ conidia per mL. Six hours after Sys application, an aliquot of 10 µL of the fungus spore suspension was applied to the leaves. The assay was carried out using four plants per treatment, which were incubated in a growth chamber at 23 ± 1 °C, for a 16 h photoperiod and under 90% RH. The size of the lesions was measured at different days post inoculums (pi). Lesion dimensions were measured using a digital caliber (Neiko 01407A).

### 3.4. Aphidius Ervi Flight Behavior

Bioassays with *Aphidius ervi (A. ervi)* parasitic wasps were conducted in wind tunnel (100 × 50 × 50 cm), as previously described in detail [44]. Plants were tested 24 h after the treatment with Sys and Scp experimental peptides (100 nM), and control buffer applied directly on leaves or added in the hydroponic growth solution. *A. ervi* naïve females, 1–2 days old, mated and fed, were released singularly in the wind tunnel, 50 cm downwind from the target plant and observed up to 5 min to determine their flight orientations and landings on the plant. Insect behavior was recorded as “Oriented flight” when the females flew within 5 cm of plant or landed on it. Similarly, it was recorded as “Landing on target” when females landed on plant. Bioassays were conducted by observing at least 100 females on 6 different plants for each treatment on 6 different days. Plants were presented in random order each day to avoid any daily bias. The experimental conditions were a temperature of 20 ± 1 °C; 65 ± 5% RH; wind speed, 25 ± 5 cm/s; Photosynthetic Photon Flux Density (PPFD) at releasing point, 700 μmol m^2^/s.

### 3.5. Volatile Organic Compounds (VOCs) Collection and Analysis

VOCs sampling and analyses were performed under controlled temperature, at 25 ± 1 °C. Leaf treated plants and control (100 pM Sys or 100 pM Scp or buffer) were used for headspace volatile collection and VOCs analysis. Headspace sampling was performed 1h after closing five plants in a glass box (60 × 60 × 60 cm) to accumulate VOCs. The collected headspaces were directly injected into the Proton Transfer Reaction ionization with a Time-of-Flight Mass Spectrometry (PTR-TOF-MS) drift tube hated (110 °C) peek inlet tube with a flow rate of 100 sccm for calculation.

VOCs were detected in real-time through proton transfer reactions using Proton Transfer Reaction-Quadrupole interface Time of Flight- Mass Spectrometry (PTR-Qi-TOF-MS) apparatus supplied by Ionicon Analytik GmbH (Innsbruck, Austria). The drift tube was kept under controlled conditions of pressure (3.8 mbar), temperature (80 °C) and voltage (1000 V), resulting in a field density ratio (E/N) of 141 Td (E being the electric field strength and N the gas number density; 1Td = 10−17 V cm^−2^).

The raw data recorded by the PTR apparatus were acquired by the TofDaq software (Tofwerk AG, Thun, Switzerland), normalized per plant and subsequently evaluated with the PTR-MS Viewer 3.2.6 (Ionicon analytic GmbH, Innsbruck, Austria).

### 3.6. Calibration of Methyl-Jasmonate Standard

The absolute quantification of methyl jasmonate (*m/z* 152.15) was performed using the IONICON Liquid Calibration Unit (LCU) coupled with PTR-Qi-TOF-MS. LCU evaporates aqueous standards into a gas stream, resulting in a gas flow containing compounds at exactly know trace concentrations. To produce a calibration curve for MeJA, a gradient flow has been obtained by nebulizing both, the liquid standard (MeJA at concentration of 10^–6^) and the distilled water, starting from 100% water to 100% MeJA. Nitrogen was utilized as a carrier gas at 1000 sccm (nitrogen with a purity of 5.0—i.e., 99.999%—purchased from Linde-Vienna-Austria) with a constant flow. The combined liquid from the two inlets was sprayed and evaporated inside the heated spray chamber at the temperature of 100 °C and was introduced in the inlet of PTR-Qi-TOF-MS. Finally, data were filtered to remove all peaks ascribed to water chemistry (*m/z* 21.022 and *m/z* 39.033 corresponding toH_3_^18^O^+^ and H_2_OeH_3_^18^O^+^, respectively) or other interfering ions (e.g., oxygen, nitrogen monoxide).

### 3.7. Gene Expression Analysis

Three fully-expanded leaves per plant were treated and three plants for each treatment (Sys or Scp or buffer) were used as biological replicates. Treated leaves and un-treated leaves of treated plants (named as distal leaves) were harvested at different time points, immediately frozen in liquid nitrogen and stored at −80 °C until use. For experiments in hydroponics, plants were grown in three different tanks and supplied with nutritive solution without (control plants) or with (treated plants) 100 pM Sys or 100 pM Scp. Three leaves per plant and three plants per each experimental condition were harvested 3 h after treatment and stored as described above. The isolation of total RNA from leaves, the synthesis of the first strand cDNA and real-time PCRs were performed according to standard procedures, as already described elsewhere [45]. For each sample, two technical replicates for each of the three biological replicates were used for the gene expression analysis. Relative quantification of gene expression was carried out using the 2^−ΔΔCt^ method [46]. The housekeeping gene EF-1α was used as endogenous reference gene for the normalization of the expression level of the target genes [47,48]. Primers and their main features are reported in Table S1.

### 3.8. Statistical Analysis

Differences in relative quantities of defense transcripts were analyzed by comparing *ΔCt* values by one-way or two-way ANalysis Of Variance (ANOVA), while for coupled comparisons a two-tailed Student’s test was used. For the insect assay, larval weights were compared by one-way ANOVA or Kruskal-Wallis non parametric ANOVA, followed by Tukey-Kramer honestly significant difference (HSD) and Dunn’s post test for multiple mean value comparisons. Survival curves of *S. littoralis* larvae and adults were compared by using Kaplan-Meier and log-rank analysis. The time required by larvae to pupate was compared by Kruskal-Wallis non parametric ANOVA followed by Dunn’s post test for multiple mean value comparisons, while the number of laid eggs was compared by one-way ANOVA, coupled with Tukey-Kramer multiple comparisons test. For the evaluation of Sys effect on *B. cinerea* infection, necrosis area differences between control and 100 pM Sys-treated sample were analyzed by T-Student’s test. Size differences of the necrotic areas, induced by fungal inocula on plants treated with Sys or Scp via root uptake, were analyzed by one-way ANOVA coupled with Tukey-Kramer honestly significant difference (HSD) test.

The number of parasitoids responding, as oriented and non-oriented flight, to each target plant was compared by a G-test for independence, as described in [49].

Differences in VOCs released by treated and control plants were compared using Kruskal-Wallis non parametric ANOVA.

## 4. Discussion

Plants have several strategies to counteract damage caused by insect and pathogens that include the induction, upon attack, of endogenous peptides, triggering defense responses against invaders. One of the major issues to address for exploiting at the best this source of molecular biodiversity for their possible use in agriculture, is the development of suitable delivery strategies of these defense molecules, which prevent environmental degradation and loss of biological activity. Here we contribute to this research area using systemin, a well-known octadecapeptide hormone of the tomato plant, that triggers plant defense pathways against different biotic stress agents and enhances defense barriers when constitutively expressed in plants [30,50,51]. Sys supply to tomato plants proved to be very effective in conferring measurable protection against *S. littoralis* and *B. cinerea*, demonstrating that both hydroponics and leaf spotting represent useful peptide delivery strategies for pest control.

Interestingly, it was recently demonstrated that the Sys peptide effectively spread throughout tomato stems and leaves following injection into the stem and leaves of tomato plants [52]. The transient pattern of gene expression registered upon Sys leaf treatment, proving that the treatment activates the octadecanoid pathway locally and systemically, is in general agreement to what expected for the early expressed defense signaling genes (*ProSys* and *AOS*), and the late downstream genes (*Pin I* and *Pin II*) encoding defensive molecules, which directly target the insect pests [11]. Our data show that *AOS* transcript increases in distal leaves after 6 h from Sys treatment. This result is apparently in contrast with the early involvement of this gene in the signaling events of the Sys-dependent defense pathway. However, since AOS is the first enzyme in the branch pathway leading to the biosynthesis of JA, the most straightforward interpretation of this result is that the enzyme contributes to the production of JA in distant leaves to trigger the systemic activation of defense related genes [53]. This hypothesis is corroborated by the high increased production of Methyl-Jasmonate (MeJA) in Sys-treated plants.

The increased expression of genes of the octadecanoid pathway leads to the production of JA and to the activation of defense genes such protease inhibitors [7]. The increased transcription of this genes is possibly associated with an increased accumulation of the inhibitors known to be involved in the reduction of growth and life of chewing herbivores and necrotrophic fungi [30]. In addition, resistance to *B. cinerea* and *S. littoralis* could be dependent on a response to Sys mediated by the pepr1/2 ortholog receptor-like kinase1, a protein with biological functions in systemin signaling and tomato immune responses [54,55].

Considering that the plant cell wall is semi-permeable, it is possible to speculate that it allows Sys to pass through and interact with its receptor with the subsequent activation of the signaling cascade.

These results are corroborated by previous observations showing that plant treatment with peptides induced defense genes and metabolites [56,57]. For example in *Arabidopsis*, 4-week-old plants grown in soil sprayed withPep1 showed an increased expression of a gene encoding a defensin [58] while plant treatment with the bacterial peptide flagellin induces the expression of numerous defense-related genes and triggers resistance to pathogenic bacteria [59]. In addition, *Solanum pimpinellifolium (S. pimpinellifolium)* roots elongated in response to systemin treatment, thus suggesting that the root perceive the peptide [60].

A number of compounds associated with indirect defense were retrieved in treated plants 10 folds in respect to controls. These compounds are known to be signals acting as synomones as they bring an advantage to both the emitter plant and the receiver organism: be it an insect (i.e., a natural enemy that finds its prey) or another plant (i.e., a neighbour unchallenged plant) [36,61,62,63]. The results of behavioral bioassay with *A. ervi* are consistent with the volatile blend released by Sys-treated plants. Among these compounds, β-caryophyllene is reported to be identified at antennal level by *A. ervi* at a concentration as low as 0.01 mg/mL and to elicit a significant higher attractiveness towards this parasitoid in respect to control solvent when tested as purified compounds in wind tunnel bioassay [64].

Surprisingly, adults *S. littoralis* of emerged from larvae fed on Sys-treated plants had reduced survival rate and lower fecundity, suggesting that Sys treatment has a strong effect on the fitness of the insect population. Plant watering with Sys solution could be an interesting option for protection against pests and pathogens, as hydroponics is largely used for tomatoes, the most widely grown vegetable in the world, and other *Solanaceae* that may benefit from Sys supply. To exploit at the best this potential, a large array of Sys concentrations should be investigating in order to assess the lowest peptide levels able to confer effective protection to tomato and other *Solanaceae* crops.

We also demonstrated that Sys treatment of healthy plants increase the attraction of *A. ervi*, a natural antagonist of the aphid *Macrosiphum euphorbiae (M. euphorbiae)*, thus inducing a reinforcement of the indirect defense barriers. Interestingly, tomato plants constitutively expressing *ProSys* were moderately tolerant to *M. euphorbiae* attacks [30]. It is tempting to speculate that, in presence of *A. ervi*, Sys treated plants will show an increased tolerance to *M. euphorbiae* attacks.

The development of safe and sustainable crop protection strategies is a challenging goal facing our society. This is increasingly pursued through bioinspired research efforts, aiming to mimic natural mechanisms of pest suppression [2]. Application of plant endogenous peptides prompting defense responses that affect the fitness and behavior of herbivores and pathogens represents a very safe approach of plant protection, due to the expected low or null toxicity of these molecules on non-target organisms. However, the evaluation of the cost of the treatment on plant physiology should be further investigated.

Although Sys homologues have been described only in solanaceous plants belonging to the subtribe Solaneae, like tomato, potato, black nightshade, and pepper [65], other genetically distinct families of plant defense signal peptides have been identified in different species reviewed in [14]. In *Arabidopsis* Pep1 is released from the C-terminus of a longer precursor protein (ProPep) and is perceived as a DAMP by specific receptors with the consequent amplification of the plant innate immune responses against pathogens. The constitutive expression of the precursor confers resistance to *Arabidopsis* plants against the oomycete plant pathogen *Pythium irregulare (P. irregulare)* [11]. Conversely, *Zea mays (Z. mays)* Pep3 regulates direct and indirect anti-herbivore defenses, likely by modulating the downstream signaling response to insect oral secretions [66]. ProPep orthologous were identified in numerous species [11] and, interestingly, a functional orthologous was also found in tomato, where it is involved in defense against a root pathogen [67]. In addition, our unpublished results suggest the presence of the recently identified systemin receptor 1 and 2 (SYR1 and SYR2), responsible of Sys perception in species of *Solanaceae*, not only in representative species of the sister subfamily *Nicotianoideae* [17], but also in other higher plants. Interestingly, the SYR and SYR-like genes are close relatives of the plant elicitor peptide receptors (PEPRs) [28,68] suggesting that the defensive signal transduction could be mediated by the same or similar players in higher plants, despite their phylogenetic distances.

## 5. Conclusions

The presented data, collectively, indicate that in different evolutionary lineages, peptides evolved as defense signals involved in the finely tuned orchestration of gene expression underlying plant immune responses. The development of control strategies of biotic stresses implying their direct delivery to the plants represents a powerful tool for sustainable agriculture that could reduce the use of chemical inputs while providing food quality and safety. This goal can be further pursued by developing bioformulations able to modulate plant defense. An example of such a formulation is represented by Messenger^®^, a Trade Mark product, that enhances disease and pest resistance in treated plants. These enhancements are based on the activity of naturally occurring proteins, the active ingredients in Messenger^®^, that trigger natural defense systems against many diseases and pests [69]. Towards this aim we are presently producing recombinant Sys in bacteria (unpublished) in order to greatly reduce the cost and increase the feasibility of the proposed approaches. In addition, despite the continuous exposition of pests to Sys within the naturally occurring tomato-pests interaction no pest’s resistance to the peptide was observed thus suggesting a good durability of the proposed approaches.

Although the involvement of Sys in the activation of tomato plant defenses was proven previously, to our knowledge this is the first work proving that that the treatment of healthy unwounded tomato plant with Sys confers resistance against pests representing a promising strategy for pest control. The effect of this peptide on multiple stress agents, both biotic and abiotic (i.e., salt tolerance) and the efficacy of different delivery strategies is very promising from an applied perspective representing a significant addition towards the field use of defense peptides in crop protection.

## Figures and Tables

**Table 1:** Volatile organic compounds (VOCs) increase upon treatment with the systemin peptide. List of VOCs significantly improved by Sys foliar application in comparison to VOCs blend released by mock- and Scp-treated plants (* *p* < 0.05, Kruskal-Wallis One Way ANOVA).

			Concentration (PPb)		
Name	Molecular Formula	Molecular Weight g/mol	Control	Sys	Scp
Benzaldehyde *	C_7_H_6_O	106.124	1.54 × 10^6^ ± 1.2 × 10^5^	3.09 × 10^6^ ± 2.8 × 10^5^	1.81 × 10^6^ ± 1.18 × 10^5^
Ethylbenzene, p-Xylene *	C_8_H_10_	106.168	1.53 × 10^6^ ± 1.43 × 10^5^	3.06 × 10^6^ ± 2.7 × 10^5^	1.81 × 10^6^ ± 1.16 × 10^5^
β-Ocimene *	C_10_H_16_	136.238	1.01 × 10^6^ ± 8.1 × 10^4^	1.22 × 10^7^ ± 1.46 × 10^5^	1.19 × 10^6^ ± 0.97 × 10^5^
α-pinene *	C_10_H_16_	136.238	1.01 × 10^6^ ± 8.1 × 10^4^	1.22 × 10^7^ ± 1.46 × 10^5^	1.19 × 10^6^ ± 0.97 × 10^5^
limonene *	C_10_H_16_	136.238	1.01 × 10^6^ ± 8.1 × 10^4^	1.22 × 10^7^ ± 1.46 × 10^5^	1.19 × 10^6^ ± 0.97 × 10^5^
Methyl Jasmonate *	C_13_H_20_O_3_	224.300	6.4 × 10^5^ ± 4.2 × 10^4^	1.16 × 10^6^ ± 5.17 × 10^4^	5.68 × 10^5^ ± 4.02 × 10^4^
β-caryophyllene *	C_15_H_24_	204.357	1.75 × 10^5^ ± 2.5 × 10^4^	0.95 × 10^6^ ± 7.6 × 10^4^	1.16 × 10^5^ ± 1.37 × 10^4^

## Supplementary Materials

The following are available online at https://www.mdpi.com/2223-7747/8/10/395/s1, Figure S1: Symptoms of *B. cinerea* infection on tomato leaves. Necrosis caused by *B. cinerea* spores 3 and 9 days post inoculums are shown in control (A, C) and Sys-treated (B, D) leaves, Figure S2: Absolute quantification of methyl-jasmonate (MeJA) released by systemin-treated plants. Standard curve and calculation of released amount of MeJA in tomato plants treated with Sys, Scp or mock on intact leaves, Figure S3: Relative quantification of defense transcripts upon Scp foliar treatment. Expression analysis of *ProSys* and *Pin I* by Real Time RT-PCR 6 h following Scp treatment. Relative quantities are calibrated on samples obtained from Red Setter leaves spotted with PBS1X. No significant differences were registered (One-way ANOVA). Error bars indicate standard error, Figure S4: Effect of 100 pM Scp added in hydroponics. Relative quantities of defense transcripts by Real Time RT-PCR detected in leaves after 3 h of hydroponics. Relative quantities are calibrated on samples obtained from tomato leaves of plant grown in a hydroponic system supplied with PBS1X. No significant differences were registered (One-way ANOVA). Error bars indicate standard error.

## References

[1] Dayan F.E., Cantrell C.L., Duke S.O. (2009). Natural products in crop protection. Bioorg. Med. Chem..

[2] Pennacchio F., Giordana B., Rao R., Beckage N., Drezen J. (2012). Applications of Parasitoid Virus and Venom Research in Agriculture. Parasitoid Viruses, Symbionts and Pathogens.

[3] Xu S., Zhou W., Pottinger S., Baldwin I.T. (2015). Herbivore associated elicitor-induced defenses are highly specific among closely related *Nicotiana* species. BMC Plant Biol..

[4] Savatin D.V., Gramegna G., Modesti V., Cervone F. (2014). Wounding in the plant tissue: The defense of a dangerous passage. Front. Plant Sci..

[5] Albersheim P., Anderson A.J. (1971). Proteins from plant cell walls inhibit polygalacturonases secreted by plant pathogens. Proc. Natl. Acad. Sci. USA.

[6] Chai H.B., Doke N. (1987). Activation of the potential of potato leaf tissue to react hypersensitively to *Phytophthora infestans* by cystospore germination fluid and the enhancement of the potential by calcium ion. Physiol. Mol. Plant Pathol..

[7] Pearce G., Strydom D., Johnson S., Ryan C.A. (1991). A polypeptide from tomato leaves induces wound-inducible proteinase inhibitor proteins. Science.

[8] Chen K., Kirber M.T., Xiao H., Yang Y., Keaney J.F. (2008). Regulation of ROS signal transduction by NADPH oxidase 4 localization. J. Cell Biol..

[9] Bergey D.R., Orozco-Cárdenas M., De Moura D.S., Ryan C.A. (1999). A wound- and systemin-inducible polygalacturonase in tomato leaves. Proc. Natl. Acad. Sci. USA.

[10] Torres M.A., Dangl J.L., Jones J.D. (2002). *Arabidopsis* gp91^phox^ homologues *AtrbohD* and *AtrbohF* are required for accumulation of reactive oxygen intermediates in the plant defense response. Proc. Natl. Acad. Sci. USA.

[11] Huffaker A., Pearce G., Ryan C.A. (2006). An endogenous peptide signal in *Arabidopsis* activates components of the innate immune response. Proc. Natl. Acad. Sci. USA.

[12] Albert M. (2013). Peptides as triggers of plant defense. J. Exp. Bot..

[13] Czyzewicz N., Yue K., Beeckman T., Smet I.D. (2013). Message in a bottle: Small signalling peptide outputs during growth and development. J. Exp. Bot..

[14] Yamaguchi Y., Barona G., Ryan C.A., Pearce G. (2011). GmPep914, an eight-amino acid peptide isolated from soybean leaves, activates defense-related genes. Plant Physiol..

[15] Ryan C.A. (2000). The systemin signalling pathway: Differential activation of plant defensive genes. Biochim. Biophys. Acta.

[16] Scheer J.M., Ryan C.A. (2002). The systemin receptor SR160 from *Lycopersicon peruvianum* is a member of the LRR receptor kinase family. Proc. Natl. Acad. Sci. USA.

[17] Wang L., Einig E., Almeida-Trapp M., Albert M., Fliegmann J., Mithöfer A., Kalbacher H., Felix G. (2018). The systemin receptor SYR1 enhances resistance of tomato against herbivorous insects. Nat. Plants.

[18] Schilmiller A.L., Howe G. (2005). Systemic signaling in the wound response. Curr. Opin. Plant Biol..

[19] Sun J., Jiang H., Li C. (2011). Systemin/Jasmonate-Mediated Systemic Defense Signaling in Tomato. Mol. Plant.

[20] Beloshistov R.E., Dreizler K., Galiullina R.A., Tuzhikov A.I., Serebryakova M.V., Reichardt S., Shaw J., Taliansky M.E., Pfannstiel J., Chichkova N.V. (2017). Phytaspase-mediated precursor processing and maturation of the wound hormone systemin. New Phytol..

[21] Scheer J.M., Pearce G., Ryan C.A. (2003). Generation of systemin signaling in tobacco by transformation with the tomato systemin receptor kinase gene. Proc. Natl. Acad. Sci. USA.

[22] Pearce G. (2011). Systemin, hydroxyproline-rich systemin and the induction of protease inhibitors. Curr. Protein Pept. Sci..

[23] Bhattacharya R., krishna Koramutla M., Negi M., Pearce G., Ryan C.A. (2013). Hydroxyproline-rich glycopeptide signals in potato elicit signalling associated with defense against insects and pathogens. Plant Sci..

[24] Narváez-Vásquez J., Orozco-Cárdenas M.L., Ryan C.A. (2007). Systemic wound signaling in tomato leaves is cooperatively regulated by systemin and hydroxyproline-rich glycopeptide signals. Plant Mol. Biol..

[25] Pearce G., Moura D.S., Stratmann J., Ryan C.A. (2001). Production of multiple plant hormones from a single polyprotein precursor. Nature.

[26] Schmelz E.A., Carroll M.J., LeClere S., Phipps S.M., Meredith J., Chourey P.S., Alborn H.T., Teal P.E. (2006). Fragments of ATP synthase mediate plant perception of insect attack. Proc. Natl. Acad. Sci. USA.

[27] Huffaker A., Dafoe N.J., Schmelz E.A. (2011). ZmPep1, an ortholog of *Arabidopsis* elicitor peptide 1, regulates maize innate immunity and enhances disease resistance. Plant Physiol..

[28] Yamaguchi Y., Pearce G., Ryan C.A. (2006). The cell surface leucine-rich repeat receptor for AtPep1, an endogenous peptide elicitor in *Arabidopsis*, is functional in transgenic tobacco cells. Proc. Natl. Acad. Sci. USA.

[29] McGurl B., Pearce G., Orozco-Cardenas M., Ryan C.A. (1992). Structure, expression, and antisense inhibition of the systemin precursor gene. Science.

[30] Coppola M., Corrado G., Coppola V., Cascone P., Martinelli R., Digilio M.C., Pennacchio F., Rao R. (2015). Prosystemin Overexpression in Tomato Enhances Resistance to Different Biotic Stresses by Activating Genes of Multiple Signaling Pathways. Plant Mol. Biol. Rep..

[31] Ahmad F.H., Wu X., Stintzi A., Schaller A., Schulze W.X. (2019). The systemin signaling cascade as derived from time course analyses of the systemin-responsive phosphoproteome. Mol. Cell Proteom..

[32] Zhang H., Yu P., Zhao J., Jiang H., Wang H., Zhu Y., Botella M.A., Samaj J., Li C., Lin J. (2018). Expression of tomato prosystemin gene in *Arabidopsis* reveals systemic translocation of its mRNA and confers necrotrophic fungal resistance. New Phytol..

[33] Chen H., Wilkerson C.G., Kuchar J.A., Phinney B.S., Howe G.A. (2005). Jasmonate-inducible plant enzymes degrade essential amino acids in the herbivore midgut. Proc. Natl. Acad. Sci. USA.

[34] Ryan C.A., Pearce G. (1998). Systemin: A polypeptide signal for plant defensive genes. Annu. Rev. Cell Dev. Biol..

[35] McGurl B., Orozco-Cárdenas M., Pearce G., Ryan C.A. (1994). Overexpression of the prosystemin gene in transgenic tomato plants generates a systemic signal that constitutively induces proteinase inhibitor synthesis. Proc. Natl. Acad. Sci. USA.

[36] Corrado G., Sasso R., Pasquariello M., Iodice L., Carretta A., Cascone P., Ariati L., Digilio M.C., Guerrieri E., Rao R. (2007). Systemin regulates both systemic and volatile signaling in tomato plants. J. Chem. Ecol..

[37] Degenhardt D.C., Refi-Hind S., Stratmann J.W., Lincoln D.E. (2010). Systemin and jasmonic acid regulate constitutive and herbivore-induced systemic volatile emissions in tomato, *Solanum lycopersicum*. Phytochemistry.

[38] Orsini F., Cascone P., De Pascale S., Barbieri G., Corrado G., Rao R., Maggio A. (2010). Systemin-dependent salinity tolerance in tomato: Evidence of specific convergence of abiotic and biotic stress responses. Physiol. Plant.

[39] Corrado G., Agrelli D., Rocco M., Basile B., Marra M., Rao R. (2011). Systemin-inducible defense against pests is costly in tomato. Biol. Plant.

[40] Coppola M., Cascone P., Madonna V., Di Lelio I., Esposito F., Avitabile C., Romanelli A., Guerrieri E., Vitiello A., Pennacchio F. (2017). Plant-to-plant communication triggered by systemin primes anti-herbivore resistance in tomato. Sci. Rep..

[41] Romanelli A., Moggio L., Montella R.C., Campiglia P., Iannaccone M., Capuano F., Pedone C., Capparelli R. (2011). Peptides from Royal Jelly: Studies on the antimicrobial activity of jelleins, jelleins analogs and synergy with temporins. J. Pept. Sci..

[42] Czyzewicz N., Stes E., De Smet I., Kleine-Vehn J., Sauer M. (2017). Tips and Tricks for Exogenous Application of Synthetic Post-translationally Modified Peptides to Plants. Plant Hormones: Methods and Protocols.

[43] Sannino L., Espinosa B., Balbiani A. (2001). Lepidotteri Delle Ortive e del Tabacco.

[44] Guerrieri E., Pennacchio F., Tremblay E. (1993). Flight behaviour of the aphid parasitoid *Aphidius ervi* (Hymenoptera: Braconidae) in response to plant and host volatiles. Eur. J. Entomol..

[45] Corrado G., Alagna F., Rocco M., Renzone G., Varricchio P., Coppola V., Coppola M., Garonna A., Baldoni L., Scaloni A. (2012). Molecular interactions between the olive and the fruit fly *Bactroceraoleae*. BMC Plant Biol..

[46] Livak K.J., Schmittgen T.D. (2001). Analysis of relative gene expression data using real-time quantitative PCR and the 2^−ΔΔ*C*_T_^ method. Methods.

[47] Marum L., Miguel A., Ricardo C.P., Miguel C. (2012). Reference Gene Selection for Quantitative Real-time PCR Normalization in Quercussuber. PLoS ONE.

[48] Müller O.A., Grau J., Thieme S., Prochaska S., Adlung N., Sorgatz A., Bonas U. (2015). Genome-Wide Identification and Validation of Reference Genes in Infected Tomato Leaves for Quantitative RT-PCR Analyses. PLoS ONE.

[49] Sokal R.R., Rohlf F.J. (1995). Biometry: The Principles and Practice of Statistics in Biological Research.

[50] Dıaz J., ten Have A., van Kan J.A. (2002). The role of ethylene and wound signaling in resistance of tomato to *Botrytis cinerea*. Plant Physiol..

[51] El Oirdi M., El Rahman T.A., Rigano L., El Hadrami A., Rodriguez M.C., Daayf F., Vojnov A., Bouarab K. (2011). *Botrytis cinerea* manipulates the antagonistic effects between immune pathways to promote disease development in tomato. Plant Cell.

[52] Mucha P., Ruczynski J., Dobkowski M., Backtrog E., Rekowski P. (2019). Capillary electrophoresis study of systemin peptides spreading in tomato plant. Electrophoresis.

[53] Chauvin A., Caldelari D., Wolfender J.L., Farmer E.E. (2013). Four 13-lipoxygenases contribute to rapid jasmonate synthesis in wounded *Arabidopsis thaliana* leaves: A role for lipoxygenase 6 in responses to long-distance wound signals. New Phytol..

[54] AbuQamar S., Chai M.F., Luo H., Song F., Mengiste T. (2008). Tomato protein kinase 1b mediates signaling of plant responses to necrotrophic fungi and insect herbivory. Plant Cell.

[55] Xu S., Liao C.J., Jaiswal N., Lee S., Yun D.J., Lee S.Y., Garvey M., Kaplan I., Mengiste T. (2018). Tomato PEPR1 ORTHOLOG RECEPTOR-LIKE KINASE1 Regulates Responses to Systemin, Necrotrophic Fungi, and Insect Herbivory. Plant Cell.

[56] de la Noval B., Pérez E., Martínez B., León O., Martínez-Gallardo N., Délano-Frier J. (2007). Exogenous systemin has a contrasting effect on disease resistance in mycorrhizal tomato (*Solanum lycopersicum*) plants infected with necrotrophic or hemibiotrophic pathogens. Mycorrhiza.

[57] Pastor V., Sánchez-Bel P., Gamir J., Pozo M.J., Flors V. (2018). Accurate and easy method for systemin quantification and examining metabolic changes under different endogenous levels. Plant Methods.

[58] Yamaguchi Y., Huffaker A., Bryan A.C., Tax F.E., Ryan C.A. (2010). PEPR2 is a second receptor for the Pep1 and Pep2 peptides and contributes to defense responses in *Arabidopsis*. Plant Cell.

[59] Zipfel C., Robatzek S., Navarro L., Oakeley E.J., Jones J.D., Felix G., Boller T. (2004). Bacterial disease resistance in *Arabidopsis* through flagellin perception. Nature.

[60] Holton N., Caño-Delgado A., Harrison K., Montoya T., Chory J., Bishop G.J. (2007). Tomato BRASSINOSTEROID INSENSITIVE1 is required for systemin-induced root elongation in *Solanum pimpinellifolium* but is not essential for wound signaling. Plant Cell.

[61] Webster B., Bruce T., Dufour S., Birkemeyer C., Birkett M., Hardie J., Pickett J. (2008). Identification of volatile compounds used in host location by the black bean aphid, Aphis fabae. J. Chem. Ecol..

[62] Song B., Liang Y., Liu S., Zhang L., Tang G., Ma T., Yao Y. (2017). Behavioral responses of *Aphis citricola* (Hemiptera: Aphididae) and its natural enemy *Harmonia axyridis* (Coleoptera: Coccinellidae) to non-host plant volatiles. Fla. Entomol..

[63] Engelberth J., Alborn H.T., Schmelz E.A., Tumlinson J.H. (2004). Airborne signals prime plants against insect herbivore attack. Proc. Natl. Acad. Sci. USA.

[64] Sasso R., Iodice L., Woodcock C.M., Pickett J.A., Guerrieri E. (2009). Electrophysiological and behavioural responses of *Aphidius ervi* (Hymenoptera: Braconidae) to tomato plant volatiles. Chemoecology.

[65] Constabel C.P., Yip L., Ryan C.A. (1998). Prosystemin from potato, black nightshade, and bell pepper: Primary structure and biological activity of predicted systemin polypeptides. Plant Mol. Biol..

[66] Huffaker A., Pearce G., Veyrat N., Erb M., Turlings T.C., Sartor R., Shen Z., Briggs S.P., Vaughan M.M., Alborn H.T. (2013). Plant elicitor peptides are conserved signals regulating direct and indirect anti herbivore defense. Proc. Natl. Acad. Sci. USA.

[67] Trivilin A.P., Hartke S., Moraes M.G. (2014). Components of different signalling pathways regulated by a new orthologue of AtPROPEP1 in tomato following infection by pathogens. Plant Pathol..

[68] Lori M., Van Verk M.C., Hander T., Schatowitz H., Klauser D., Flury P., Gehring C.A., Boller T., Bartels S. (2015). Evolutionary divergence of the plant elicitor peptides (Peps) and their receptors: Interfamily incompatibility of perception but compatibility of downstream signalling. J. Exp. Bot..

[69] Wei Z., Betz F.S. (2007). Messenger^®^: An Environmentally Sound Solution for Crop Production and Protection. ACS Symp. Ser..

