# Supplementary figures and images for: Tomato Plants Treated with Systemin Peptide Show Enhanced Levels of Direct and Indirect Defense Associated with Increased Expression of Defense-Related Genes
Source: Plants (Basel). 2019 Oct 3;8(10):395. doi: 10.3390/plants8100395 (PMC6843623; doi:10.3390/plants8100395)

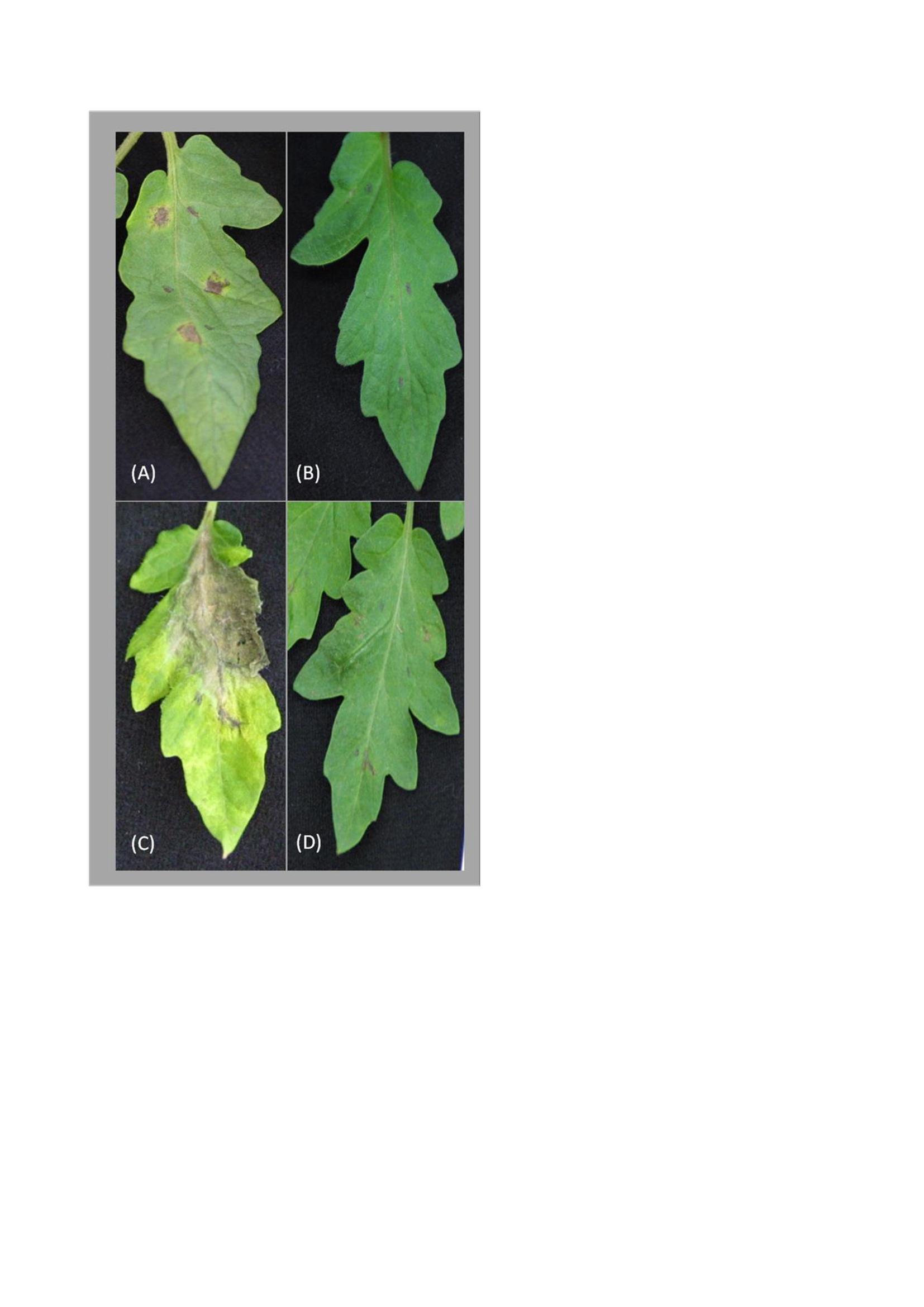

Supplement: Supplementary file 1 [file plants-08-00395-s001.zip › Figure-S1.tiff]

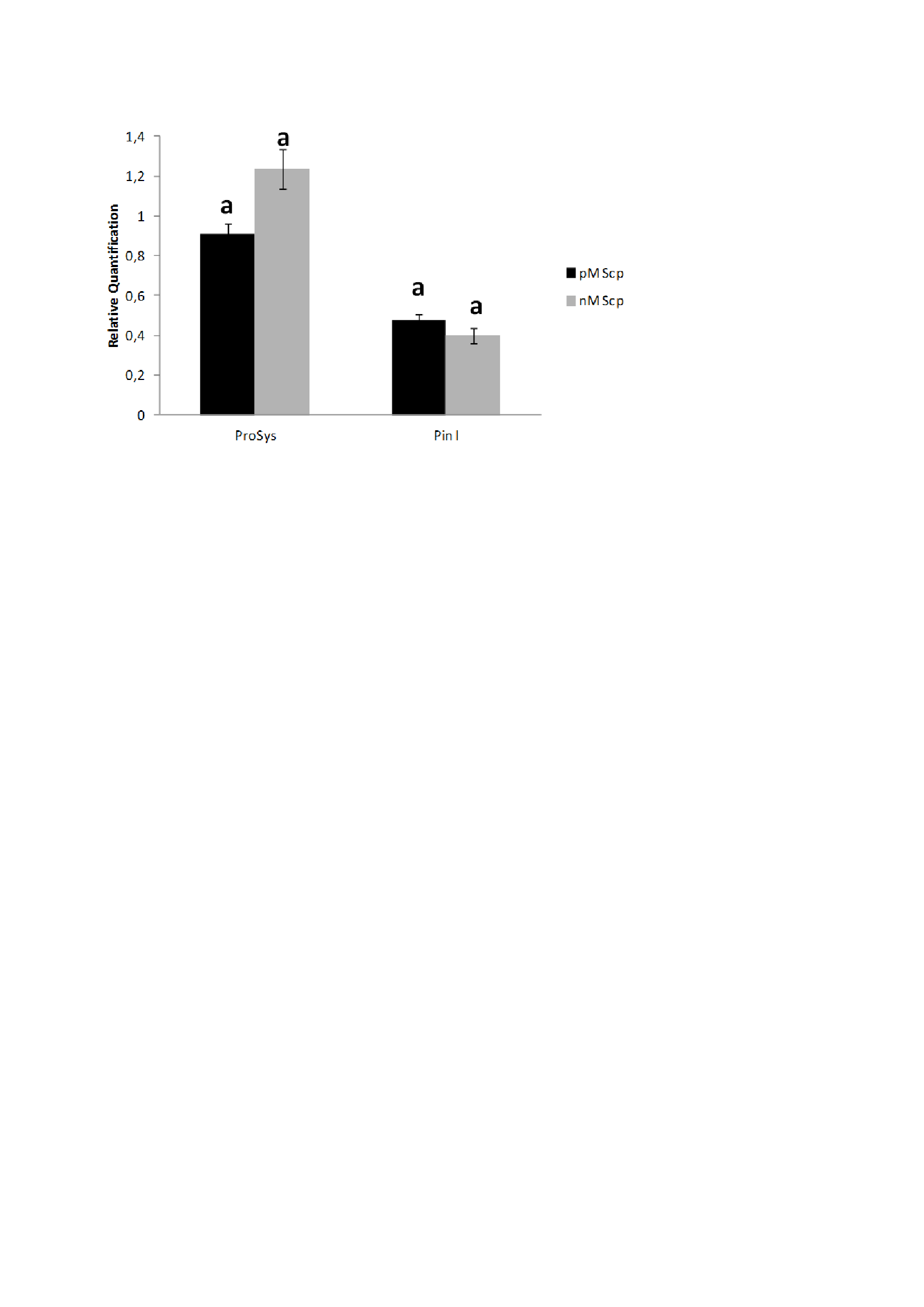

Supplement: Supplementary file 1 [file plants-08-00395-s001.zip › Figure-S2.tiff]

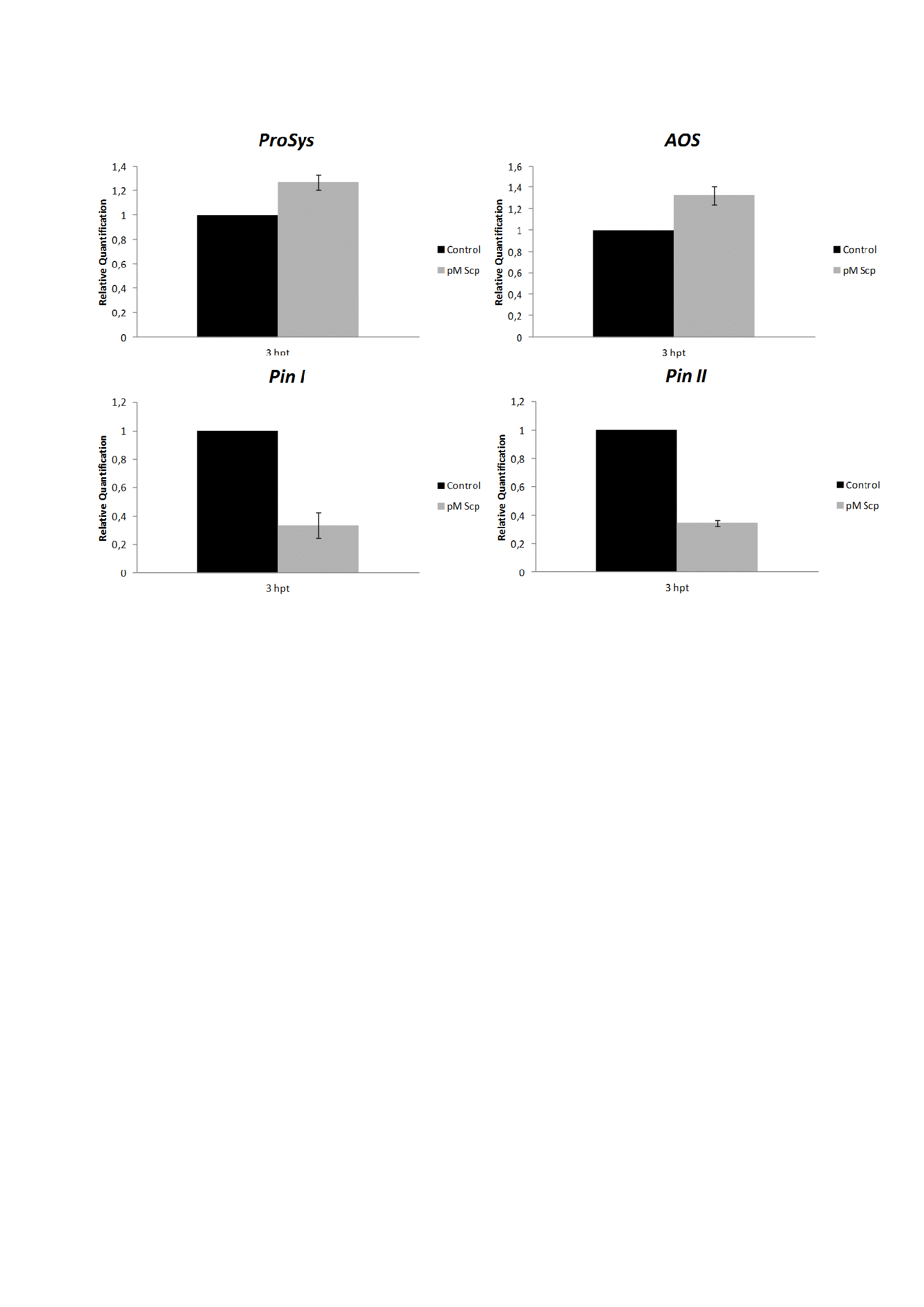

Supplement: Supplementary file 1 [file plants-08-00395-s001.zip › Figure-S3.tiff]
